# Self-perception of oral health in older adults from an urban population in Lisbon, Portugal

**DOI:** 10.1590/S1518-8787.2016050006311

**Published:** 2016-08-16

**Authors:** Catarina Carvalho, Ana Cristina Manso, Ana Escoval, Francisco Salvado, Carla Nunes

**Affiliations:** I Escola Nacional de Saúde Pública. Universidade Nova de Lisboa. Lisboa, Portugal; II Instituto Superior de Ciências da Saúde Egas Moniz. Centro de Investigação Interdisciplinar Egas Moniz. Caparica, Portugal

**Keywords:** Older Adults, Self-Assessment, Oral Health, Socioeconomic Factors, Cross-Sectional Studies

## Abstract

**OBJECTIVE:**

To analyze if the self-perception of oral health in the urban context is associated with sociodemographic factors that interfere in the life quality of oral health.

**METHODS:**

Cross-sectional study with convenience sample of older individuals (65 years old or more) enrolled in the *Agrupamento de Centros de Saúde de Lisboa Norte* (ACES Lisboa Norte – Health Centers Groupings North Lisbon). The self-perception of oral health and associated life quality was evaluated by the Geriatric Oral Health Assessment Index and the individuals were classified according to sociodemographic characteristics. The internal consistency of the questionnaire was evaluated by Cronbach’s alpha (α). Later, we used binary logistic regression models to characterize the factors associated with the self-perception of oral health, considering the sociodemographic variables and the older adults’ clinical conditions of oral health and establishing the crude and adjusted (to age) odds ratios and their 90% confidence intervals.

**RESULTS:**

A total of 369 older adults participated in this study, with an average age of 74.2 years (SD = 6.75); 62.9% were female. On average, the index was moderated, with tendency to be high: 32.9 (SD = 3.6; 12-36 interval). The Cronbach’s alpha was high: 0.805. Age, marital status, and the last dental appointment were the factors significantly associated with self-perception of oral health.

**CONCLUSIONS:**

The study shows that these individuals have a moderate, with tendency to high, self-perception of oral health. The self-perception of oral health assessment allowed us to identify the main associated sociodemographic factors. This instrument can help guiding planning strategies and oral health promotion directed toward a better life quality for this population group.

## RESUMO

**OBJETIVO:**

Analisar se a autopercepção de saúde bucal em contexto urbano está associada aos factores sociodemográficos que interferem na qualidade de vida da saúde bucal.

**MÉTODOS:**

Estudo transversal com amostra de conveniência de indivíduos idosos (65 anos ou mais) inscritos no Agrupamento de Centros de Saúde de Lisboa Norte. A autopercepção da saúde bucal e qualidade de vida associada foi avaliada pelo índice de avaliação da saúde bucal em idosos (*Geriatric Oral Health Assessment Index)* e os indivíduos foram classificados de acordo com as características sociodemográficas. A consistência interna do questionário foi avaliada por meio do alfa (α) de Cronbach. Posteriormente, foram utilizados modelos de regressão logística binária para caracterizar os factores associados com a autopercepção de saúde bucal considerando as variáveis sociodemográficas e de condições clínicas de saúde bucal dos idosos e determinados os *odds ratios* bruto e ajustado (à idade) e respectivos intervalos de confiança a 90%.

**RESULTADOS:**

Participaram 369 idosos, com média de idade de 74,2 anos (DP = 6,75); 62,9% eram do sexo feminino. Em média, o índice foi moderado com tendência a elevado: 32,9 (DP = 3,6; intervalo 12-36). O alfa de Cronbach foi elevado: 0,805. A idade, o estado civil e a última consulta de Medicina Dentária foram os factores significativamente associados a autopercepção da saúde bucal.

**CONCLUSÕES:**

O estudo mostra que esses indivíduos apresentam uma autopercepção de saúde bucal moderada, com tendência a elevada. A avaliação da autopercepção da saúde bucal permitiu identificar os principais factores sociodemográficos associados. Este instrumento pode contribuir para orientar as estratégias de planejamento e promoção da saúde bucal direcionadas para uma melhor qualidade de vida deste grupo populacional.

## INTRODUCTION

According to the United Nations^a^, the aging of the world population is a lasting phenomenon with global characteristics, affecting the whole society at all levels and requiring deep social changes.

In Portugal, population aging emphasizes the need for public health policies that focus on the maintenance of a satisfactory life quality at that age. According to the 2011 Censuses, Lisbon presents high level of aging, coming out from 103 older adults per 100 young people, in 2001, to 140 older adults per 100 young people in 2011, and is, at the same time, the city with lowest percentage of young people (12.9%)^b^.

Population aging increases the growing need to set acceptable standards of oral health to contribute to a better general health and well-being state^12^. To this end, we need epidemiological data that quantify the oral health conditions of older adults. This type of information is essential to the planning, organization, and monitoring of the provided oral health services^22^.

The literature suggests that, in addition to clinical data of evaluation by health professionals, data should be collected on indicators of self-perception, especially relating to oral health conditions and treatment needs perceived by older adults^22^. In this sense, various sociodental indicators have been developed to measure how oral health changes compromise the life quality and well-being of individuals. This includes subjective measures such as pain or discomfort, aesthetic problems, restrictions on feeding, communication, affective relationships, daily activities, and physical and psychological well-being of individuals^11^. From the instruments developed to evaluate these indicators, Oral Health Impact Profile (OHIP), Oral Impacts on Daily Performances (OIDP), Oral Health-Related Quality of Life (OHRQOL), and Geriatric Oral Health Assessment Index (GOHAI) stand out^3^. The latter was developed specifically for the older adults’ population^4^.

The aim of this study was to analyze if the self-perception of oral health in the urban context is associated with sociodemographic factors that interfere in the life quality of oral health.

## MATERIAL AND METHODS

### Health Service in Portugal

In Portugal, the Ministry of Health is responsible for the development of health policies, as well as for the management of the National Health Service (NHS). There are five regional health administrations (ARS) – ARS Alentejo, ARS Algarve, ARS Centro, ARS Lisbon and Tagus Valley, and ARS North – responsible for the execution of the goals of the national health policies, for the development of guidelines and protocols, and for monitoring health care. Decentralization efforts have focused their goals in changing the financial and management responsibility to the regional level. In practice, however, the autonomy of the regional health authorities is limited to the primary health care. The autonomous regions of Azores and Madeira have effective autonomy in regional health strategies and in the management of budget and expenses^c^.

The ARS Lisbon and Tagus Valley is in charge of 15 Health Centers Groupings (ACES). They are: ACES North Lisbon, Central Lisbon, West Lisbon and Oeiras, Cascais, Amadora, Sintra, Loures-Odivelas, Tagus Estuary, Almada-Seixal, Arco Ribeirinho, Arrabida, North West, South West, Middle Tagus, and Wet Area.

The oral health care system in Portugal, a mixture of coexistence between the Beveridgian, Bismarckian, and Private or Liberal models, is strongly conditioned by the low coverage of the NHS and is characterized by three coverage systems: the liberal coverage system or private insurance, the social security system, and the system financed by taxes^10^.

### Sample

Cross-sectional study, with non-probabilistic and convenience sample of 369 individuals, corresponding to 5.1% margin of error for a 90% confidence level. Individuals aged 65 years or more were selected, randomly, among those who expected the appointment at the health center, between February 2012 and June 2013. We considered the listing of individuals enrolled in 2008, in the Northern Unit of Lisbon – older adults users of the North Lisbon health centers, which have as reference the Hospitals of Santa Maria and Pulido Valente.

This group consists of five health centers: Alvalade, Benfica, Coração de Jesus, Lumiar, and Sete Rios, in which 295,687 users were registered in 2012, with 62,498 older adults.

The interview was conducted face-to-face by four examiners, properly calibrated and trained according to the biosafety regulations, under the environmental conditions existing in the health centers. We included older people aged more than 65 years, with ability to respond to the questionnaire, regulars at the North Lisbon health centers, who accepted to participate in the study (informed consent).

### Data Collection Instrument

The applied questionnaire consisted of three distinct parts. The first one covered the sociodemographic characterization, composed by age, occupation, situation regarding employment, place of birth, marital status, education level, household income, sex, ability of life autonomy, among others. The second included questions about the older adults’ oral health habits, such as: when the last visit to the dentist took place, if they received information on how to do mouth examination to prevent oral cancer, if they use prosthesis, and how they clean it. In the third part, we applied the Geriatric Oral Health Assessment Survey Index (GOHAI) to evaluate the self-perception of oral health, index translated and validated to the Portuguese language^6^.

The GOHAI consists of 12 questions, related with the influence of oral health problems in the following physical, psychosocial, and pain or discomfort dimensions:

- The physical function, represented by the pattern of mastication, speech, and swallowing^1^ ^,^ ^23^
- The psychosocial function, represented by the concern about oral health, satisfaction or dissatisfaction with appearance, self-awareness about their oral health, and by avoiding social contact because of oral problems^1^ ^,^ ^13^ ^,^ ^23^
- The pain or discomfort, represented by the use of medication to relieve pain or discomfort^1^ ^,^ ^13^ ^,^ ^23^

For Atchison and Dolan^4^, the response options can have from three to six categories (from “always” to “never”). In this study we opted for the simplified frequency scale suggested by the authors, with three categories: “always”, “sometimes”, and “never” (values of 1, 2, and 3, respectively).

To obtain the final index, we conducted the simple sum of the values, in a scale of 12 to 36. The GOHAI index classifies the self-perception in “high” (34 to 36 points), “moderate” (30 to 33 points) and “low” (< 30 pontos), by the Atchison and Dolan^4^ criteria for simplified scale.

### Data analysis

The dependent variable considered was the older adults’ self-perception in relation to oral health; the independent ones were the sociodemographic variables and older adults’ clinical conditions of oral health.

We used descriptive statistics measures (for the general characterization of data) and determined the Cronbach’s alpha (α) (for assessing the internal consistency of the questionnaire). Later, we used binary logistic regression models to characterize the factors associated with the self-perception of low oral health of the older adults, considering the sociodemographic variables (sex, age, marital status, educational level, family income, and ability of life autonomy) and clinical conditions of oral health (last dentist appointment and prosthesis use). We determined the crude and adjusted by age odds ratios and their 90% confidence intervals. A significance level of 5% was used.

### Ethical remarks

This study was approved by the Ethics Committee of the Direcção Geral da Saúde of the Portuguese Ministry of Health (Process 210, December 17, 2010). All participants signed the informed consent form.

## RESULTS

A total of 369 older adults participated in the study, which correspond to 3/5 of the older population enrolled in this group. They were aged in average 74.2 years (SD = 6.75); 62.9% were female, and 37.1% male. The vast majority was retired (95.0%); 53.9% presented education level up to four years, and 87.8% considered themselves totally independent regarding their daily activities. Only 13.0% knew that there is an oral health professional in the health center to which they belong; 51.1% did not visit a dentist for over a year; 62.6% of individuals used dental prosthesis, and 68.0% were satisfied with it (Table 1).

**Table 1:** Sociodemographic and clinic characteristics distributed according to the categories of self-perception defined by GOHAI. Health Centers North Lisbon, 2012-2013.

Sociodemographic characteristics	n	%	GOHAI					

			Low self-perception of oral health (< 30)		Moderate self-perception of oral health (30-33)		High self-perception of oral health (34-36)	
		
			n	%	n	%	n	%
Sex								
Male	137	37.1	20	14.6	34	24.8	83	60.6
Female	232	62.9	33	14.2	69	29.7	130	56.0
Age group (years)								
65-74	202	54.7	24	11.9	59	29.2	119	58.9
75-84	136	36.9	25	18.4	32	23.5	79	58.1
≥ 85	31	8.4	4	12.9	12	38.7	15	48.4
Marital status								
Married or cohabitation	222	60.2	31	14.0	64	28.8	127	57.2
De facto separated or divorced	28	7.6	3	10.7	7	25.0	18	64.3
Single	12	3.3	4	33.3	2	16.7	6	50.0
Widower	107	29.0	15	14.0	30	28.0	62	57.9
Education level								
Cannot read nor write	31	8.4	6	19.3	11	35.5	14	45.2
Can read and write	20	5,4	5	25.0	4	20.0	11	55.0
4th year	228	61.8	32	14.0	67	29.4	129	56.6
9th year	44	11.9	4	9.1	11	25.0	29	65.9
12th year	30	8.1	4	13.3	8	26.7	18	60.0
Higher Education	16	4.3	2	12.5	2	12.5	12	75.0
Household income								
< 1 minimum wage	112	33.3	19	17.0	31	27.6	62	55.4
1 to 2 minimum wages	166	49.4	26	15.7	49	29.5	91	54.8
2 to 4 minimum wages	50	14.9	5	10.0	13	26.0	32	64.0
> 4 minimum wages	8	2.4	1	12.5	3	37.5	4	50.0
Ability of life autonomy								
Totally independent	324	87.8	44	13.6	87	26.8	193	59.6
Dependent	45	12.2	9	20.0	16	35.6	20	44.4
Clinical conditions								
Last dental appointment								
Less than a year ago	142	38.6	29	20.4	36	25.4	77	54.2
More than a year ago	188	51.1	19	10.1	58	30.9	111	59.0
Uses prosthesis								
Yes	231	62.6	33	14.3	66	28.6	132	57.1
No	138	37.4	20	14.5	37	26.8	81	58.7

GOHAI: Geriatric Oral Health Assessment Index

The application of GOHAI resulted in mean values close to the upper limit of the measure variation scale for the global index (mean = 32.9; SD = 3.6) and for its three dimensions, being possible to say that the older adults favorably evaluated their oral health (Table 2). The majority (57.7%) of individuals showed high self-perception of oral health, with values greater than 33; 27.9% showed moderate self-perception (values between 30 and 33); and only 14.4%, low self-perception (values below 30).

**Table 2:** Older adults’ self-perception of oral health: mean, confidence interval, and GOHAI variation amplitude. Health Centers North Lisbon, 2012-2013.

Variable	Mean	90%CI	Variation amplitude
GOHAI	32.85	32.5–33.2	16 to 36
Physical dimension	10.7	10.6–10.9	4 to 12
Psychosocial dimension	8.4	8.3–8.5	4 to 9
Pain or discomfort dimension	13.7	13.5–13.8	6 to 15

To determine the internal consistency of the questionnaire, we used the Cronbach’s alpha (α), obtaining a value of α = 0.805. Individuals aged 75-84 years have 1.670 (90%CI 1.003–2.783; p = 0.098) more probability to present self-perception of low oral health than older adults with 65-74 years. The older, with more than 85 years, have equal probability of presenting self-perception of low oral health than individuals with 65-74 years (Table 3).

**Table 3:** Factors associated with the older adults’ self-perception of low oral health. Health Centers North Lisbon, 2012-2013.

Variable	OR_crude_	90%CI	p	OR_adjusted_ to age	90%CI	p
Age (years)			0.245			
65-74*						
75-84	1.670	1.003–2.783	0.098			
≥ 85	1.099	0.424–2.844	0.871			
Marital status			0.320			0.255
Married or cohabitation*						
De facto separated or divorced	0.739	0.258–2.122	0.638	0.698	0.242–2.015	0.577
Single	3.081	1.071–8.860	0.080	3.221	1.110–9.349	0.071
Widower	1.005	0.575–1.755	0.989	0.883	0.494–1.577	0.724
Last dental appointment			0.033			0.026
More than a year ago*						
Less than a year ago	2.283	1.350–3.859	0.010	2.361	1.391–4.009	0.008
Does not know	1.348	0.557–3.262	0.579	1.345	0.554–3.268	0.583

* Reference class.

Regarding the last dental appointment, older adults who had the appointment less than a year ago have 2.283 (90%CI 1.350–3.859; p = 0.010) more probability to present self-perception of low oral health than individuals who had the appointment more than a year ago.

Single individuals, 3.221 (90%CI 1.110–9.349; p = 0.071), are more likely to present self-perception of low oral health than individuals married or in cohabitation, after adjusted to age.

Regarding the last dental appointment, individuals who had the appointment less than a year ago have 2.361 (90%CI 1.391–4.009; p = 0.010) more probability to present self-perception of low oral health than individuals who had the appointment more than a year ago, after adjusted to age.

## DISCUSSION

Older adults, aged 65 years old or more, enrolled in the Health Centers Grouping North Lisbon, present a self-perception of moderate, with tendency to high, oral health. From the studied sociodemographic variables, only age, marital status, and the last dental appointment were factors significantly associated with self-perception of oral health.

Currently, there is an increasing interest in studies of self-perception of health for knowing and monitoring it, and studies of this nature are increasingly recommended by the World Health Organization (WHO)^18^. This study came to add new data to this field, hitherto little explored in Portugal.

The average age found among the older adults of this study was 74.15 years, and this value is close to the most recent data on the Portuguese population of older adults^d^ and also to the average found in other studies, which ranged between 67.1 and 73.5 years^1^ ^,^ ^22^. More than half of the sample consisted of women, and the 2011 Censuses help to explain these results, because they indicate that the preponderance of the female population is enhanced as age increases, due to the higher life expectancy at birth of women.

As to the education level, most individuals had only the fourth year of schooling, which corresponds to the data of the 2001 Labor Inquiry, which found that the older adults’ population holds, generally, low levels of education^d^. The household income is a really important variable in the analysis of the older adults’ health situation. In this study, approximately 50.0% of the sample has incomes between one and two minimum wages, being these data according to the data provided by Eurostat^e^.

The high percentage of individuals entirely independent may be due to the fact that the study has been performed in health centers, where they go with the capacity to do so.

The average of the GOHAI values in this study (32.9) suggested moderate self-perception of oral health of the population under study, although the analysis by classes refers to a higher perception. With median 34.0 and mode 36.0, we consider that the population of this study has a moderate, with tendency to high, perception, as in the original version of the GOHAI^1^ and in other studies^5^, in China, Japan^16^, and Arabia^2^. Ribeiro et al., in 2012, justify these results insofar as the older adults consider their oral health condition as good, although they have many dental losses and significant oral amendments. This apparent contradiction is explained by the lack of information in this age group in taking naturally that aging is associated with inevitable disabilities, such as tooth loss^20^.

The questionnaire presented good internal consistency (α = 0.81), indicating the validity of all questions. This also occurred in the original version in English^1^, which obtained Cronbach’s alpha coefficient of 0.79, and in the Portuguese version, with alpha of 0.768^5^. The internal consistency was evaluated in several other countries (Spain, China, France, Sweden, Malaysia, Japan, Germany, Turkey, Jordan, and Mexico)^13^, obtaining á values between 0.75 (in Turkey) and 0.92 (in Germany)^21^.

The most affected domain of GOHAI was the psychosocial one, suggesting that the older adults have limited their contact with other people because of the appearance of their teeth. Furtado et al.^8^, in 2011, and Cárdenas et al.^4^, in 2012, also found that the psychosocial domain is the most affected one, mainly because the older adults feel uncomfortable to eat in front of others because of their teeth problems. Fonseca et al.^7^, in 2011, observed that the most affected domain was the physical or functional one, especially in the discomfort in swallowing food. The fact that senior citizens can express themselves, share joys and anxieties, talk with others about a part of life, enjoy food, and be nourished by them are very important performances^8^.

Regarding the age group, younger seniors (aged 65-74 years) showed greater self-awareness of their problems in oral health. In fact, Nunes et al.^17^, in 2012, showed that advancing age contributed to worsen the self-perception of the oral health state, noting greater tendency for values of self-perception of low of oral health in individuals between 75-84 years. Miranda et al.^15^, in 2011, found better results in seniors over 75 years old, and the authors relate these values to the older adults’ adaptation process or the recognition of the deterioration of health conditions as normal in aging.

As for marital status, single individuals were the ones who showed lower values of self-perception, similar results to other studies^15^ ^,^ ^17^ ^-^ ^19^. This may be due to the high impact of loneliness on life quality, suggesting that individuals who live alone may present lower emotional stability which, in turn, induces a low perception level^9^.

Regarding the last dental appointment, individuals who had the last appointment less than a year ago have a higher tendency to present lower values of self-perception of oral health. In fact, older adults tend to visit a dentist only when they have oral problems associated with pain or discomfort^14^. Still, those visits are determined by bad past experiences or phobias, which could generate a vicious cycle: less visits can exacerbate problems of oral health. On the other hand, this tendency toward a lower self-perception of oral health may be related to a greater awareness of older adults regarding their oral health, considering the recent visit to their dentist^6^.

It is still likely that the low association of these variables in relation to the self-perception of oral health can be explained by the low prevalence of a dentist visit in the previous year. This may reflect the lack of access to the services of medical-dental assistance, which in Portugal are mostly private^3^.

Regarding the limitations of this study, one of them is the fact of not carrying out clinical examination with the questionnaire, which should be implemented in the future, making it possible to evaluate the behavior of the clinical conditions of the oral cavity in relation to the self-perception of each one and vice versa. Additionally, the sample was of convenience and of relatively small size, which can condition the representativeness of the studied population. For example, the fact that there are usually more women in health centers and that they naturally have an increased availability to take part, can condition the representativeness of the sample^6^ ^,^ ^19^. To obtain more solid and useful conclusions for society, it is necessary to carry out more comprehensive and representative studies and with a larger number of participants.

This study represents a starting point so that we can know better the oral health conditions of the older users of primary health care. The study also shows the usefulness of GOHAI to guide the planning strategies and oral health promotion directed toward a better life quality of this population group.

In this sense, it is important to educate about oral health, instilling prevention measures, which, in addition to reducing the problems, help to aware every one about their real needs for treatment.

## Footnotes

a United Nations, Population Division, Departmenet of Economic and Social Affairs. Old-age dependency ratio 65+/(20-64) by major area, region and country, 1950-2100 (ratio of population 65+ per 100 population 20-64). New York; 2011.

b Instituto Nacional de Estatística. Censos 2011: resultados definitivos - Região Lisboa. Lisboa; 2013 [cited 2014 Feb 16]. Available from: http://migre.me/uFEvS

c Direcção-Geral da Saúde (PT), Direcção de Serviços de Epidemiologia e Estatísticas de Saúde, Divisão de Estatísticas de Saúde. Elementos estatísticos: informação geral: Saúde 2008. Lisboa; 2008.

d Instituto Nacional de Estatística. Censos 2011: resultados definitivos - Região Lisboa. Lisboa; 2013 [cited 2014 Feb 16]. Available from: http://migre.me/uFEaw

e EUROSTAT. Population structure and ageing. Luxembourg: European Comission; 2010 [cited 2015 Oct 14]. Available from: http://migre.me/uFE92
